# Case report and literature review: A *de novo* pathogenic missense variant in *ACTN4* gene caused rapid progression to end-stage renal disease

**DOI:** 10.3389/fped.2022.930258

**Published:** 2022-08-25

**Authors:** Zhechi He, Ke Wu, Wenqing Xie, Jianghua Chen

**Affiliations:** ^1^Kidney Disease Center, The First Affiliated Hospital, Zhejiang University School of Medicine, Hangzhou, China; ^2^Prenatal Diagnosis Center, Yiwu Maternity and Child Health Care Hospital, Yiwu, China

**Keywords:** focal segmental glomerulosclerosis, end-stage renal disease, proteinuria, *ACTN4*, *de novo*

## Abstract

**Background:**

Focal segmental glomerulosclerosis (FSGS) is a histopathological diagnosis of the sclerosis of glomeruli and the damage to renal podocytes. FSGS affects the filtration function of the kidneys and results in nephrotic syndrome (NS) in children and adults. FSGS is a clinically and genetically heterogeneous disorder. FSGS-1 [OMIM #603278] is one of the progressive hereditary renal diseases. It is caused by heterozygous variants of the *actinin alpha 4* (*ACTN4*) [OMIM*604638] gene on chromosome 19q13.2 in a dominant inheritance (AD) manner. With the recent development of whole-exome sequencing (WES), 22 (including our case) pathogenic or likely pathogenic variants have been identified in *ACTN4* gene.

**Case presentation:**

We reported a 17-year-old Chinese girl who was hospitalized with foamy urine, nausea and vomiting. Laboratory tests revealed increased levels of serum creatinine and urea nitrogen. Ultrasonography demonstrated bilaterally reduced size of kidneys. The primary diagnoses were NS and chronic kidney disease stage 5 (CKD5). The hemodialysis was initiated in 48 h after admission. After 4 months of treatment, the patient received an allogeneic kidney transplantation from her father. A novel heterozygous missense variant c.494C > T (p.A165V) in the *ACTN4* gene was found by WES in the patient. This variant was confirmed by Sanger sequencing. The computational simulation of the stability of mutant protein (p.A165V) was decreased. Interatomic interactions of the p.A165V site were increased, and it might be associated with the increased ubiquitylation in the vicinity of the mutant site.

**Conclusion:**

As per the guidelines of the American College of Medical Genetics and Genomics for interpreting sequence variants, the novel heterozygous missense variant was pathogenic (PS2 + PM1 + PM2 + PP3 + PP4). It should be noted that the early onset of severe proteinuria with a poor prognosis is an important and universal symptom for most genetic FSGS. If necessary, genetic screening is recommended.

## Introduction

Focal segmental glomerulosclerosis (FSGS) is a pathological feature of glomerular diseases that presents clinically with proteinuria and a progressive decline in renal function. Patients with FSGS may have initial symptoms of nephrotic syndrome (NS), such as massive proteinuria, hypoalbuminemia, hyperlipidemia, hypertension, and sepsis. FSGS1–FSGS10 are genetically heterogeneous disorders. They are caused by variants in *ACTN4* (AD FSGS1 [OMIM #603278]), *TRPC6* (AD FSGS2 [OMIM #603965]), *CD2AP* (susceptibility to FSGS3 [OMIM #607832]), *APOL1* (susceptibility to FSGS4 [OMIM #612551]), *INF2* (susceptibility to FSGS5 [OMIM #613237]), *MYO1E* (AR FSGS6 [OMIM #614131]), *PAX2* (AD FSGS7 [OMIM #616002]), *ANLN* (AD FSGS8 [OMIM #616032]), *CRB2* (AD FSGS9 [OMIM #616220]), and *LMX1B* (AD FSGS10 [OMIM #256020]), respectively. Pathogenic variants in the *ACTN4* gene are rarely detected in individuals with NS or severe proteinuria. Sadowski et al. (1) showed that disease-causing *ACTN4* variants were not found in 2016 individuals with steroid-resistant nephrotic syndrome (SRNS). In addition, Wang et al. (2) also did not detect *ACTN4* variants in 110 Chinese children with SRNS. Nagano et al. (3) found that only 2 of 230 Japanese patients with severe proteinuria had *ACTN4* variants. Following a review of literature, only 21 pathogenic or likely pathogenic *ACTN4* variants were identified. Most variants within the actin binding domain (ABD) of ACTN4 protein contributed to cytoskeleton and podocytes *via* increasing the binding affinity of ACTN4 to filamentous-actin (F-actin) (4).

In this study, we reported a Chinese girl with a novel pathogenic missense *ACTN4* variant affected with end-stage renal disease (ESRD). We conducted a systematic literature review to summarize previously reported clinical phenotypes and *ACTN4* variants. Most cases are affected by severe proteinuria at early onset, hypertension, and podocytopathy features.

## Case presentation

A 17-year-old girl self-reported nocturia that progressed for half a year. Before being hospitalized, she had noticed foamy urine for 3 weeks. She was admitted following 5 days of nausea and vomiting. Blood tests revealed increased serum creatinine of 18.02 mg/dl, high urea nitrogen, low hemoglobin of 75 g/L, low serum albumin of 28.2 g/L, and severely increased parathyroid hormone (PTH) of 606.0 pg/ml. A lipid panel blood test revealed that the levels of triglyceride (TG), total cholesterol (TC), high-density lipoprotein cholesterol (HDL-C), and low-density lipoprotein cholesterol (LDL-C) were normal, and the level of very-low-density lipoprotein (VLDL) was decreased a bit. A urine analysis showed an increased level of proteinuria of 10.85/L/day, a positive result of a hematuria blood test, and a high level of pro-B-type natriuretic peptide (>9,000 pg/ml). In the meantime, there was no significant change in her weight. She did not has mental development delay. Ultrasonography revealed the reduced size of bilateral kidneys with hyperechoic reflections inside the renal parenchyma. A kidney biopsy was offered, but it was refused by her parents. Echocardiography indicated small accumulations of pericardial fluid, the mildly regurgitant tricuspid valve, and a slightly enlarged left ventricle. The primary diagnoses were NS, CKD5, renal anemia, and secondary hyperparathyroidism. After admission, the hemodialysis was initiated in 48 h and continued for 4 months. Then, the patient received an allogeneic kidney transplant from her father.

**FIGURE 1:**
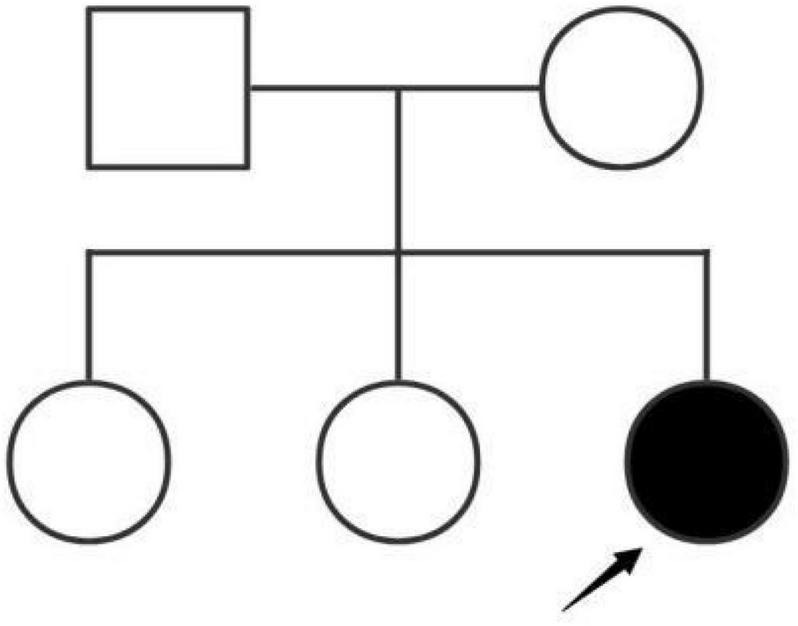
The Chinese patient’s pedigree.

The patient was the third child of phenotypically normal Chinese parents. The other two siblings were healthy and proteinuria was not found (Figure 1). Her parents did not have consanguineous relation and family history was not remarkable. She was naturally conceived with an uneventful perinatal period. She was born at term at 40 weeks of gestational age. Based on the fact that disease-causing gene variants have been identified in adolescents with idiopathic NS, genetic screening in the patient’s family for inherited diseases was recommended.

### Genetic testing

The patient and her parents signed informed consent for genetic analysis. Our legal ethics committee approved this genetic study. Genomic DNA was extracted from the peripheral blood of the patient and phenotypically normal parents for WES. Sanger sequencing was used for further verification. We identified a *de novo* heterozygous missense variant c.494C > T in the *ACTN4* gene (NM_004924.6). This variant has not been registered in population databases (1,000 Genomes Project, gnomAD, and dbSNP) or reported in disease databases (ClinVar, Human Gene Mutation Database, OMIM).

**FIGURE 2:**
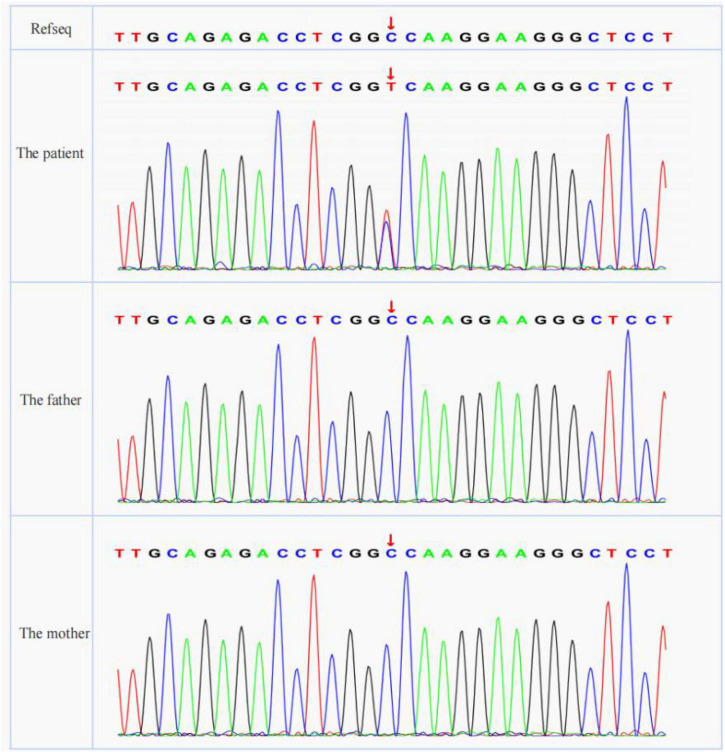
The results of Sanger sequencing (*ACTN4* variant was marked with red arrows).

**FIGURE 3:**
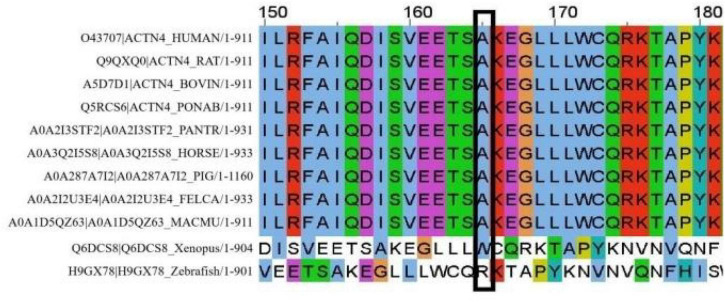
In total, 30 amino acids surrounding the variant position (marked with a black box).

**FIGURE 4:**
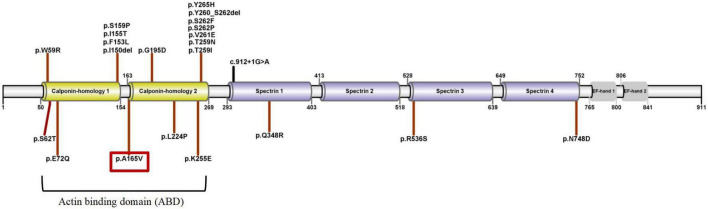
The schematic diagram of *ACTN4* variants and ACTN4 protein’s domains (UniProtKB-O43707). The patient’s *ACTN4* variant was marked with a red box.

A cosegregation analysis was performed among family members. This variant was *de novo* and verified by Sanger sequencing (Figure 2). Silico predictive algorithms (SIFT, PolyPhen-2, REVEL, MutPred, MutationTaster, PROVEAN, and MVP) of pathogenicity all showed damage. The analysis of conserved sequences suggested this variant was located in highly conserved sequences across several mammalian species (Figure 3). The variant c.494C > T (p.A165V) was located in a critical and well-established functional domain (the calponin-homology domain of ACTN4 protein) without benign variants (Figure 4). Summing up the above, according to the guidelines of the American College of Medical Genetics and Genomics (ACMG) for interpreting sequence variants, the novel missense variant was pathogenic (PS2 + PM1 + PM2 + PP3 + PP4).

### The computational simulation of the stability and interatomic interactions of mutant protein

**FIGURE 5:**
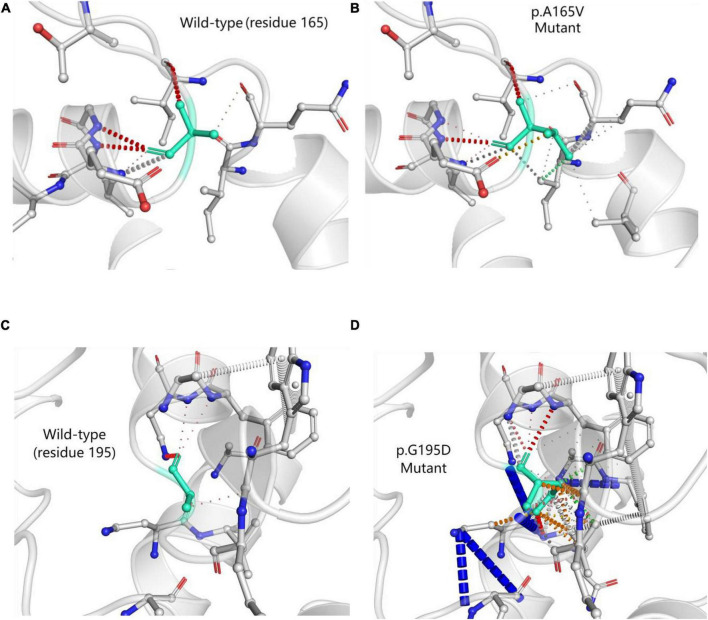
Prediction of interatomic interactions of p.A165V **(A,B)** and p.G195D **(C,D)**. Residues 165 and 195 in the wild-type and mutant proteins (p.A165V and p.G195D) are colored in light green and are shown as sticks. The respective chemical interactions are labeled as dotted lines and colored as follows: hydrogen bonds—(red), weak hydrogen bonds—(orange), hydrophobic contacts—(green), amide-amide contacts—(blue), and ionic interactions—(gold). Amino acid residues are also colored according to type, namely; nitrogen (blue), oxygen (red), and sulfur (yellow). In comparison with wild-type sites, some interactions were observed to be added in mutant sites.

DynaMut (a web server) (5) is a well-established normal mode approach. We used it to visualize and assess the stability and interatomic interactions of mutant proteins. We put information into DynaMut as follows: wild-type structure (PDB accession code: 6O31), mutation detail (A165V and chain A). The prediction outcome of stability was ΔΔG: −0.087 kcal/mol (destabilizing). In comparison with p.A165V, we put p.G195D into DynaMut. The prediction outcome of stability of p.G195D was ΔΔG: −1.349 kcal/mol (destabilizing). The residues 165 and 195 in the wild-type were observed to form residue interactions (colored in light green) with their surrounding residues, whereas some interactions were seen to be increased in mutant sites (p.A165V and p.G195D) (Figure 5).

## Literature review

We searched the PubMed database, the Human Gene Variant Database (HGMD), and Online Mendelian Inheritance in Man (OMIM) using “focal segmental glomerulosclerosis” and “*ACTN4*” as keywords. The search time was from the establishment of the databases to April 1, 2022. Previous studies with *ACTN4* variants and clinical characteristics were reviewed. In total, fifteen documents were retrieved. Although 41 mutations were registered in HGMD, we reclassified the pathogenicity of these variants according to standards and guidelines for the interpretation of sequence variants. A total of 22 pathogenic or likely pathogenic *ACTN4* variants and related phenotypes are summarized in Table 1 and Figure 4. Three heterozygous variants [c.1-34C > T, c.1-590delA, and (1-1044delT) + (1-797T > C) + (1-769A > G)] in the promoter of the *ACTN4* gene have been reported in patients with FSGS. The functional analysis of these promoter variants indicated that they might also contribute to the pathophysiology of idiopathic FSGS (6). The remaining 17 known variants are shown in Table 2. More importantly, variants in the *ACTN4* gene were missense and occurred *de novo*. Most patients with *ACTN4* variants had proteinuria at adolescent onset and rapidly progressed to ESRD.

**TABLE 1:** Genotypes and phenotypes of patients with *ACTN4* variants previously reported.

References	Ethnicity	Gender	Family history	Age of onset	Initial symptoms or diagnosis	Histopathology diagnosis	Time to ESKD	Age of kidney transplant	*ACTN4* (NM_004924.6)	Origin of variant	Protein domain	Evidence of pathogenicity	Pathogenicity
Weins et al. (7)	Western European	M	FH	5	Proteinuria	FSGS	3 y	10	c.175T > C (p.W59R)	*de novo*	ABD	PS2, PS3, PM1, PM2, PP3, PP4	Pathogenic
Dai et al. (8)	Chinese	ND	S	ND	ND	FSGS	ND	ND	c.184T > A (p.S62T)	*de novo*	ABD	PS2, PM1, PM2, PP3, PP4	Pathogenic
Weins et al. (7)	ND	ND	ND	ND	Proteinuria	FSGS	ND	ND	c.214G > C (p.E72Q)	ND	ABD	PM1, PM2, PP3, PP4	Likely pathogenic
Rodrigues et al. (5)	Colombian	ND	ND	ND	ND	FSGS	ND	ND	c.448_450delATC (p.I150del)	ND	ABD	PM1, PM2, PM4, PP3, PP4	Likely pathogenic
Barua et al. (9)	ND	ND	ND	ND	Proteinuria	FSGS	ND	ND	c.457T > C (p.F153L)	ND	ABD	PM1, PM2, PP3, PP4	Likely pathogenic
Giglio et al. (10)	Caucasian	F	S	3	SRNS	FSGS	ND	ND	c.464T > C (p.I155T)	*de novo*	ABD	PS2, PM1, PM2, PP3, PP4	Pathogenic
Bezdíčka et al. (11)	Czech	F	ND	15.1	SRNS	ND	ND	ND	c.475 T > C (p.S159P)	ND	ABD	PM1,PM2, PP3, PP4	Likely pathogenic
Bartram et al. (12)	German	F	S	13	ESKD	ND	ND	14	c.584G > A (p.G195D)	*de novo*	ABD	PS2, PS3, PM1, PM2, PP3, PP4	Pathogenic
Nagano et al. (3)	Japanese	M	ND	6	Proteinuria	FSGS	7 y	ND	c.671T > C (p.L224P)	*de novo*	ABD	PS2, PM1, PM2, PP3, PP4	Pathogenic
Kaplan et al. (13)	Oklahoma	ND	ND	ND	ND	FSGS	ND	ND	c.763A > G (p.K255E)	ND	ABD	PS3, PM1, PM2, PP3, PP4	Pathogenic
Bartram et al. (12)	Canary Islands	ND	ND	ND	ND	FSGS	ND	ND	c.776C > T (p.T259I)	ND	ABD	PS3, PM1, PM2, PP3, PP4	Pathogenic
Sen et al. (14)	ND	M	ND	6	SRNS	FSGS	ND	ND	c.776C > A (p.T259N)	ND	ABD	PM1, PM2, PM5, PP3, PP4	Likely pathogenic
Dai et al. (8)	Caucasian	F	S	7	SRNS	FSGS	8 y, 9 m	11	c.782T > A (p.V261E)	*de novo*	ABD	PS2, PM1, PM2, PP3, PP4	Pathogenic
Kakajiwala et al. (15)	Caucasian	F	S	5	NS	FSGS	6 m	6	c.784T > C (p.S262P)	*de novo*	ABD	PS2, PM1, PM2, PP3, PP4	Pathogenic
Choi et al. (16)	Korean	M	S	3	Proteinuria	FSGS	ND	ND	c.785C > T (p.S262F)	Germline mosaicism from the father’s sperm	ABD	PM1, PM2, PM5, PP3, PP4	Likely pathogenic
		F		3.7	Proteinuria	FSGS	ND	5.7					
Bierzynska et al. (17)	White	M	S	12	SRNS	FSGS	1 y, 4 m	ND	c.778_786del TATGTGTCC (p.Y260_S262del)	ND	ABD	PM1, PM2, PM4, PP3, PP4	Likely pathogenic
Feng et al. (4)	European American	F	S	14	NS	FSGS	6 m	16	c.793T > C (p.Y265H)	ND	ABD	PS3, PM1, PM2, PP3, PP4	Pathogenic
Nagano et al. (3)	Japanese	M	ND	8	Proteinuria	FSGS	ND	ND	c.912 + 1G > A	ND	-	PM2, PM6, PP3, PP4	Likely pathogenic
Rodrigues et al. (5)	Black	F	S	20	Proteinuria	FSGS	ND	ND	c.1043A > G (p.Q348R)	ND	Spectrin	PS3, PM2, PM6, PP3, PP4	Pathogenic
Schapiro et al. (18)	Kurd	M	ND	ND	Proteinuria, hematuria	FSGS	ND	ND	c.1606C > A (p.R536S)	ND	Spectrin	PM2, PM6 PP3, PP4	Likely pathogenic
Safaříková et al. (19)	Czech	F	FH	54	NS	FSGS	ND	ND	c.2242A > G (p.N748D)	ND	Spectrin	PM2, PM6 PP3, PP4	Likely pathogenic
Our patient	Chinese	F	S	17	Proteinuria	ND	ND	17	c.494C > T (p.A165V)	*de novo*	ABD	PS2, PM1, PM2, PP3, PP4	Pathogenic

M, male; F, female; y, years; m, month; FH, family history; S, sporadic; ESKD, end-stage kidney disease; ND, not done or no data; ABD, actin binding domain; FSGS, focal segmental glomerulosclerosis; NS, nephrotic syndrome; SRNS, steroid-resistant nephrotic syndrome; PS2, *de novo* (both maternity and paternity confirmed) in a patient with the disease and no family history; PS3, well-established in vitro or in vivo functional studies supportive of a damaging effect on the gene or gene product; PM1, variants located in a mutational hot spot and/or critical and well-established functional domain (e.g., active site of an enzyme) without benign variation; PM2, variants were absent from controls; PM4, protein length changes as a result of in-frame deletions/insertions in a non-repeat region or stop-loss variants; PM5, novel missense change at an amino acid residue where a different missense change determined to be pathogenic has been seen before; PM6, assumed *de novo*, but without confirmation of paternity and maternity; PP3, multiple lines of computational evidence support a deleterious effect on the gene or gene product; PP4, patients’ phenotype or family history is highly specific for a disease with a single genetic etiology.

**TABLE 2:** The known variants of VUS in the *ACTN4* gene.

*ACTN4* (NM_004924.6)	Patients’ diagnosis	Evidence of pathogenicity	Pathogenicity	References
c.459C > G (p.F153L)	Unknown	PM1, PM2, PP3	VUS	PMID: 33226606
c.485-7T > C	Developmental disorder	PM2, BP4	VUS	PMID: 28135719
c.734-26C > G	SRNS	PM2, BP4	VUS	PMID: 29869118
c.845G > A (p.C282Y) c.928C > T (p.R310W)	Bronchopulmonary dysplasia	PM2, PP3	VUS	PMID: 31848748
c.929G > A (p.R310Q)	FSGS	PS3, BP4, BP6	VUS	PMID: 21680739
c.1279G > A (p.A427T)	SRNS	PM2, BP4	VUS	PMID: 29869118
c.1660A > G (p.M554V)	Autism spectrum disorder	PS2, PM2, BP4	VUS	PMID: 25363768
c.2020C > T (p.R674C)	SRNS	PM2, PP3	VUS	PMID: 25903641
c.2084G > A (p.R695H)	SRNS	PM2, BP4	VUS	PMID: 28780565
c.2351C > T (p.A784V) c.2360C > T (p.P787L)	IgA nephropathy	PM2, BP4	VUS	PMID: 23890478
c.2378G > A (p.C793Y) c.2393G > A (p.G798D)	IgA nephropathy	PM2, PP3	VUS	
c.2401G > A (p.p.V801M)	FSGS	PM2, BP4, BP6	VUS	PMID: 24130771
c.2620G > A (p.D874N)	SRNS	PM2, BP4,	VUS	PMID: 30406062
c.2629G > A (p.E877K)	SRNS	PM2, PP3	VUS	PMID: 28780565

FSGS, focal segmental glomerulosclerosis; SRNS, steroid-resistant nephrotic syndrome; PVS1, null variant (nonsense, frameshift, canonical ± 1 or 2 splice sites, initiation codon, single or multiexon deletion) in a gene where LOF is a known mechanism of disease; PS2, *de novo* (both maternity and paternity confirmed) in a patient with the disease and no family history; PS3, well-established *in vitro* or *in vivo* functional studies supportive of a damaging effect on the gene or gene product; PM1, variants located in a mutational hot spot and/or critical and well-established functional domain (e.g., active site of an enzyme) without benign variation; PM2, variants were absent from controls; PM4, protein length changes as a result of in-frame deletions/insertions in a non-repeat region or stop-loss variants; PP3, multiple lines of computational evidence support a deleterious effect on the gene or gene product; PP5, reputable source recently reports variant as pathogenic, but the evidence is not available to the laboratory to perform an independent evaluation; BP4, multiple lines of computational evidence suggest no impact on gene or gene product (conservation, evolutionary, splicing impact, etc.); BP6, reputable source recently reports variant as benign, but the evidence is not available to the laboratory to perform an independent evaluation; CKD, chronic kidney disease; VUS, variants of unknown significance.

## Discussion

The human *ACTN4* gene is located on chromosome 19q13.2. The transcript of *ACTN4* (NM_004924.6) has 21 exons, a transcript length of 4,990 base pairs, and a translation length of 911 amino acids. The alpha-actinin-4 protein (ACTN4) [UniProtKB-O43707] is one member of four actin-binding proteins. ACTN4 is ubiquitously expressed in different tissues and highly expressed in the placenta and kidney (20). ACTN4 was prominently distributed in podocytes *via* immunostaining followed by confocal microscopy (21).

Actinin alpha 4 has three main domains: (1) the N-terminal highly conserved ABD consists of two calponin homology (CH1/2); (2) spectrin 1-4; and (3) the C-terminal domain consists of EF-hand 1/2 (Figure 4).

Most of the pathogenic or likely pathogenic variants were missense and located within ABD. *ACTN4* variants in ABD damaged the podocyte foot process architecture and function through a “gain-of-function” mechanism (increased the binding of ACTN4 to F-actin). It was a “dominant effect” that mutant ACTN4 protein unconventionally aggregated in the cell and the binding affinity of ACTN4 to F-actin was increased (22). Bartram et al. (12) identified p.G195D in a patient with juvenile onset of proteinuria and ESRD. A functional analysis of p.G195D showed that ACTN4 was the significantly reduced protein in the patient’s primary renal epithelial cells. Further analysis revealed a “dominant effect” in which the decreased stability of mutant protein was associated with the increased ubiquitylation in the vicinity of p.G195D site. The aggregated unstable mutant protein led to the disturbance of the renal cell cytoskeleton. The p.G195D and p.A165V were all located within CH2 domain of ACTN4. So we hypothesized that p.A165V may have the same pathogenic mechanism as p.G195D. The computational simulation of p.G195D seemed to accord closely with the functional analysis of p.G195D. All results showed that the stability of the mutant proteins (p.G195D and p.G195D) was decreased. The stability of the mutant protein might be mediated by the increased ubiquitylation. Therefore, it was tempting to speculate that the increased ubiquitylation in the vicinity of mutant sites may be connected with increased interatomic interactions of mutant sites. Presumably, p.A165V and p.G195D had a basic similarity in the pathogenic mechanism.

Three variants (Table 1) occurred in spectrin domains that were known to mediate CLP36-binding. Functional studies showed *ACTN4* variants in spectrin domains provided a plausible “loss-of-function” mechanism (decreased the binding of ACTN4 to CLP36). Liu et al. (23) reported that deficiencies of ACTN4 and CLP36 were observed in podocytes of FSGS cases by performing immunohistochemical staining. The mutant protein (p.Q348R) significantly inhibited the ability of ACTN4 to interact with CLP36. Depletion of the level of CLP36 or disruption of the ACTN4-CLP36 complex significantly impeded RhoA activity and the generation of traction force in podocytes but did not alter the actin-binding affinity (12).

The C-terminal EF-hand domain is involved in binding intracellular Ca^2+^. ACTN4 has two isoforms (Ca^2+^-sensitive and Ca^2+^-insensitive). The Ca^2+^-sensitive isoform is broadly expressed, while the Ca^2+^-insensitive isoform is predominantly expressed in the nervous system (24). The binding of Ca^2+^ to EF-hand domains reduced the affinity of F-actin binding (25). Five variants in HGMD occurred in EF-hand domains. Safaříková et al. (19) found p.A784V, p.P787L, p.C793Y, and p.G798D in patients with IgA nephropathy. We reclassified these four variants as uncertain significance. Chatterjee et al. (26) reported p.V801M in a patient with proteinuria. It was reclassified as a variant of uncertain significance. For now, no pathogenic or likely pathogenic variants have been identified in the C-terminal EF-hand domains.

To our knowledge, *ACTN4* variants in literature have all occurred *de novo* (except no data were collected). *ACTN4* variants within ABD showed a “gain-of-function” mechanism. The homozygous *Actn4* knockout (*Actn4* KO) mice developed albuminuria and FSGS at about 10 weeks of age, loss of Actn4 damaged glomerular podocytes and filtration barrier (13). A plausible “loss-of-function” mechanism of *ACTN4* variants within spectrin or EF-hand domains also existed. The extent of the reduction of ACTN4 may be closely associated with the severity of proteinuria in FSGS cases. As shown in Table 1, patients (14/16) with *ACTN4* variants within ABD were affected with proteinuria before the age of 18 years and developed rapidly progression to ESRD. Patients (2/16) with *ACTN4* variants within spectrin domains appeared proteinuria over 18 years old (one case was 54 years old). It needs more cases to verify whether or not two different mechanisms are associated with the age of onset.

In total, 17 known *ACTN4* variants were of unknown significance (Table 2). As per the guidelines of ACMG for interpreting sequence variants, although these variants were absent from normal controls, multiple silico predictive algorithms suggested no impact on these variants (conservation, evolutionary, splicing impact, etc.) or reputable source recently reported these variants as likely benign or benign variants. The phenotypes of some patients with these variants were not associated with FSGS. This summary (Table 2) might note that phenotypic variability of *ACTN4*-mediated diseases. Whereas, it is restricted to a small amount of reported cases. So, clinicians should be cautious of dealing with these variants.

The genetic screening is recommended for those juveniles who are accidentally found to have severe proteinuria. Although we are realistic that no treatment is available for *ACTN4*-mediated FSGS, a genetic screening can help physicians and patients to prepare the kidney transplantation in time. If parents consider to give birth to another child, a definitive genetic diagnosis is essential for prenatal diagnosis.

In conclusion, we described a Chinese patient with a novel pathogenic *ACTN4* variant. The genetic screening of congenital proteinuria is necessary for patients. We summarized the pathogenicity of *ACTN4* variants in previous literature. The severe proteinuria at early onset and rapid progression to ESRD are universal and important symptoms of *ACTN4*-mediated FSGS, and those patients are good candidates for the kidney transplantation.

## Data availability statement

The datasets for this article are not publicly available due to concerns regarding participant/patient anonymity. Requests to access the datasets should be directed to the corresponding author.

## Ethics statement

The studies involving human participants were reviewed and approved by the Ethical Committee of The First Affiliated Hospital, Zhejiang University School of Medicine. Written informed consent was obtained from the individual(s), and minor(s)’ legal guardian/next of kin, for the publication of any potentially identifiable images or data included in this article.

## Author contributions

KW wrote the main manuscript text and critically revised the manuscript. WX carried out the molecular genetic experimental. ZH and JC prepared the clinical data and imaging data. ZH and KW contributed to the check of revision, genetic evaluation, and genetic databases analysis. All authors reviewed the manuscript and read and approved the final manuscript.
